# Ameliorative effect of different mesoporous bioactive glass materials in experimental tibial defects in rats

**DOI:** 10.2478/abm-2022-0027

**Published:** 2023-06-16

**Authors:** Ozlem Ozmen, Fatma Tomul, Yusuf Sinan Sirin

**Affiliations:** Department of Pathology, Faculty of Veterinary Medicine, Burdur Mehmet Akif Ersoy University, Istiklal Yerleskesi, 15030 Burdur, Turkey; Department of Chemistry, Faculty of Art and Science, Burdur Mehmet Akif Ersoy University, Istiklal Yerleskesi, 15030 Burdur, Turkey; Department of Surgery, Faculty of Veterinary Medicine, Burdur Mehmet Akif Ersoy University, Istiklal Yerleskesi, 15030 Burdur, Turkey

**Keywords:** bone defect, immunohistochemistry, mesoporous bioactive glass, pathology, rat

## Abstract

**Background:**

Enhancing the bone healing procedure would resultantly improve the post-recovery life quality, as well as the speed with which the patient returns to their former life quality. Porous structures can provide a large surface area and abundant channels to facilitate mass transfer.

**Objective:**

To evaluate the application of mesoporous materials in the bone healing of surgically created defects on the tibiae of male adult Wistar rats.

**Methods:**

The defect areas were evaluated after implantation of 4 types of bioactive glass histopathologically and immunohistochemically. Fifty adult rats were divided into 5 groups including a control group without material. The used products were mesoporous bioactive glass (MBG), Cu-MBG, Zn-MBG, and Cu–Zn-MBG. Unicortical bone defects with a 3 mm diameter were performed in both tibiae of the animals and filled with 4 types of glass particles. The rats were then euthanized at 15 d and 30 d. Tibial samples were collected and the tissues forwarded for histological processing, and examined using light microscopy. Additionally, bone healing was evaluated by assessing the levels of bone morphogenetic protein BMP2, collagen 1, osteocalcin (OST), and vascular endothelial growth factor (VEGF) using immunohistochemical methods.

**Results:**

Within the 15th day, all groups presented connective tissue septa; at the 30th day, the new bone formation was more intense in the Cu–Zn-MBG group. Additionally, BMP2, collagen 1, OST, and VEGF immune expression were more prominent in the Cu–Zn-MBG group.

**Conclusions:**

The study results indicated that MBG may be used for the repairing of bone defects. Cu–Zn-MBG may be the best choice for this purpose.

Bone tissue has a number of important functions in the body including support for movements, protection of internal organs, acid–base buffering, calcium homeostasis, and production of blood cells [1, 2]. Bones have a high regeneration capacity and most of the small bone damages including fractures heal spontaneously. But large bone defects generally require medical or surgical treatments. For that reason, these lesions are called critical-size defects (CSDs) [2,3,4]. A CSD has been defined as a segmental bone deficiency of a length exceeding 2–2.5 times the diameter of the affected bone [5]. Treatment of these defects and regeneration of such large bone defects require the use of bone grafts to replace the damaged bone using an organic or non-organic graft material [6, 7].

Tissue engineering (TE) is one of the best solutions that considered an alternative solution for the healing of CSD. The target of the TE strategies is to prepare a bridging material (scaffold) to guide the rapid and effective regeneration of the new bone (NB) formation [8, 9]. An ideal bone graft material or scaffold for bone tissue engineering (BTE) should be osteoinductive, osteogenic, osteoconductive, biocompatible, degradable, or resorbable, and have strong mechanical properties that are similar to those of bone at the defect and implant site, to be able to provide temporary mechanical support [1, 6].

Recently, biomaterials have been used for rapid and better healing of bone defects. These materials of BTE must also be angiogenic and capable of inducing the formation of new vessels [2, 3]. Additionally, the morphology and structure of the scaffold is also important to achieve ideal bone regeneration. An excellent bone graft material should act as a bridging structure and template to guide NB tissue regeneration to take place in an optimal manner [8].

Today, mesoporous bioactive glasses (MBGs) are considered as biomaterials with a high potential for application in bone tissue regeneration due to their properties of ordered mesopores arrangements, narrow pore size distribution, and huge surface areas and pore volumes. MBGs belong to the family of bioactive glasses that have attracted much attention because of their capability to bond with bone and to stimulate osteogenesis and angiogenesis [10, 11]. MBGs can be doped during manufacture with trace amounts of elements such as strontium (Sr), zinc (Zn), and copper (Cu) that are known to promote healthy bone growth with angiogenesis [12,13,14,15]. Bioactive glasses have a compressive elastic modulus and offer strength and support for the regeneration of cortical and trabecular bone [12, 16, 17]. The present study aimed to evaluate the effects of application of different materials such as MBG, Cu-MBG, Zn-MBG, and Cu–Zn-MBG in remedying tibial bone defects in rats.

## Materials and methods

### Chemicals

In the synthesis of MBGs and metal loaded mesoporous bioactive glasses (M-MBGs), the following chemicals were used (analytical purity grade): poly(ethylene glycol)-block-poly(propylene glycol)-block-poly (ethylene glycol) (Pluronic P123, C_4_H_5_O(C_2_H_4_O)x(C_3_H_6_O)y(C_2_H_4_O)zC_4_H_5_O_2_), tetraethyl orthosilicate (TEOS, Si(OC_2_H_5_)4), triethyl phosphate (TEP, (C_2_H_5_O)_3_PO), calcium nitrate (Ca(NO_3_)_2_ 4H_2_O), copper nitrate (Cu(NO_3_)_2_ 6H_2_O), zinc nitrate (Zn(NO_3_)_2_ 6H_2_O), nitric acid (HNO_3_, 65%), and sodium hydroxide (NaOH). All chemicals were procured from Sigma-Aldrich and used without any further purification. Ultrapure water was used for the preparation of all solutions.

### Synthesis of MBG and M-MBG

MBGs were synthesized using the hydrothermal synthesis method by making some modifications to the prescription of Anand et al. [18]. In a selection that is typical of manufacturing processes applicable to MBG and M-MBG, P123 was chosen as the structure-directing agent, and TEOS and TEP, Ca(NO_3_)_2_ 4H_2_O, Cu(NO_3_)_2_ 6H_2_O, and Zn(NO_3_)_2_ 6H_2_O were chosen as the sources of Si, P, Ca, Cu, and Zn, respectively. During synthesis, 1 g of P123 was dissolved in 50 mL of a 1.6 M HNO_3_ solution at room temperature and mixed for 30 min. Then, 10 mL TEOS was added dropwise and the resulting mixture was kept in an ultrasonic bath for 30 min; TEP and calcium nitrate, at a Ca:Si:P ratio of 33:61:6 by weight (%), were added at intervals of 30 min and kept for 12 h with stirring. During the synthesis of copper loaded glasses, zinc loaded glasses, or both, the same processes as in the synthesis of calcium silicate were followed. While for the synthesis of copper loaded glasses (Cu:Ca:Si:P) and zinc loaded glasses (Zn:Ca:Si:P) separately, the copper nitrate and zinc nitrate were added at the ratio of 3:30:61:6 by weight (%), respectively, for the synthesis of copper zinc loaded glasses (Zn:Cu:Ca:Si:P), the copper nitrate and zinc nitrate were added at the ratio of 1:2:30:61:6 by weight (%) after the addition of calcium nitrate. These prepared mixtures were kept at 100°C for 48 h and then dried at room temperature. After drying, the bioactive materials were subject to a 2-step heat treatment process, consisting of the following: heating to 400°C at a 2°C/min heating rate and maintaining for 2 h at this temperature, and heating to 650°C at a 2°C/min heating rate and calcining for 4 h at this temperature. The products obtained were coded as MBG, Cu-MBG, Zn-MBG, and Cu–Zn-MBG, respectively.

All MBGs were investigated using X-ray diffraction (XRD) to identify the phases formed in the material’s surface before and after immersion in simulated body fluid (SBF). XRD patterns were obtained using a Rigaku Ultima IV diffractometer with Ni filtered CuKα (λ = 0.154,06 nm) radiation in the range of 2θ = 10º–90º, step size 0.020º, 45 kV, and 40 mA. Fourier transformed infrared (FTIR) spectra have been used to identify compounds in MBGs. FTIR spectra were obtained using the PerkinElmer Frontier model spectrometer by applying the KBr pellet technique in the range 4000–400/cm.

### In-vitro bioactivity of bioactive glasses

The in-vitro bioactivity of the synthesized materials was evaluated based on the formation of hydroxyapatite on their surface during the process of soaking them for 28 d in the SBF (pH 7.4, T = 37°C, and volume/mass ratio corresponding to VSBF/mMBGs = 200 mL/g) prepared according to the procedure proposed by Kokubo and Takadama [19]. Samples soaking in SBF were washed with water after separating from SBF by filtration and dried at 37°C for 24 h. XRD and FTIR analyzes were performed to confirm the formation of hydroxyapatite.

### Animals

Fifty 5-month-old male Wistar albino rats weighing 300–350 g were obtained from the Experimental Animal Production and Experimental Research Center of Burdur Mehmet Akif Ersoy University (Turkey). All experiments were performed in accordance with the Animal Research: Reporting in Vivo Experiments (ARRIVE) guidelines 2.0 [21], and the study approved by the Local Ethical Committee on Animal Research of Burdur Mehmet Akif Ersoy University, Turkey (certificate of approval no. 526).

The rats were kept under standard laboratory conditions (humidity 60 ± 5%; temperature 21 ± 2°C; 12:12 h light:dark cycle). All animals were fed with a standard commercial chow diet (Korkuteli Yem) and they had free access to food pellets and water.

**Figure 1.**
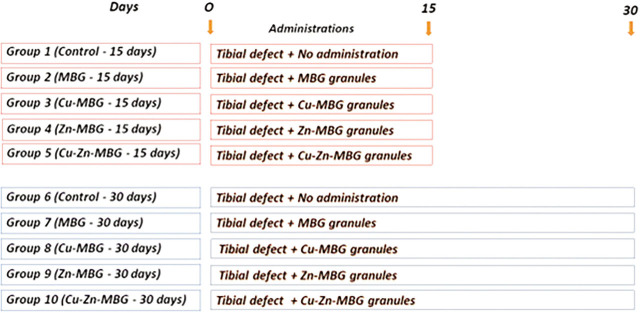
Experimental design and applications.

**Figure 2.**
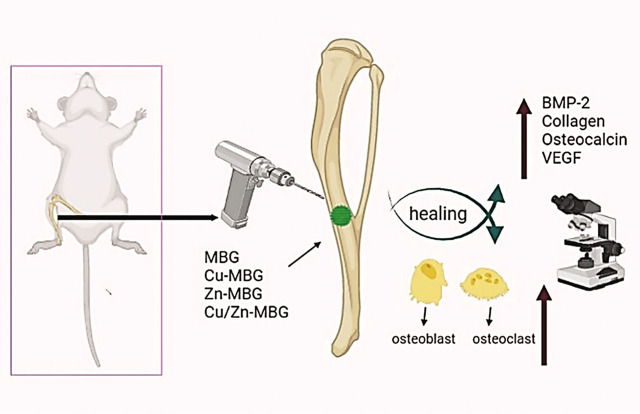
Schematic illustration of the study.

The rats were randomly distributed into 5 groups of 10 rats each and simple random sampling was used to select the rats to be assigned to each group. All rats that were comprised in these 5 groups were assigned to either of 2 groups depending on the duration of the experimentation period elapsed, and humanely euthanized at the end of the experiment. Totally 10 subgroups were used for this study. Tibial defects were created at both tibiae’s distal region, and inserted using different types of granules, which were MBG, Cu-MBG, Zn-MBG, or Cu–Zn-MBG. The animals were humanely euthanized on the 15th and 30th days post operation. Sham treatment groups were also constituted, comprised of rats with tibial defects. Totally 10 tibiae were harvested from each group. The procedures applied to the groups are shown in **Figure 1** and experimental procedures illustrated in **Figure 2**.

### Surgical procedure

**Figure 3.**
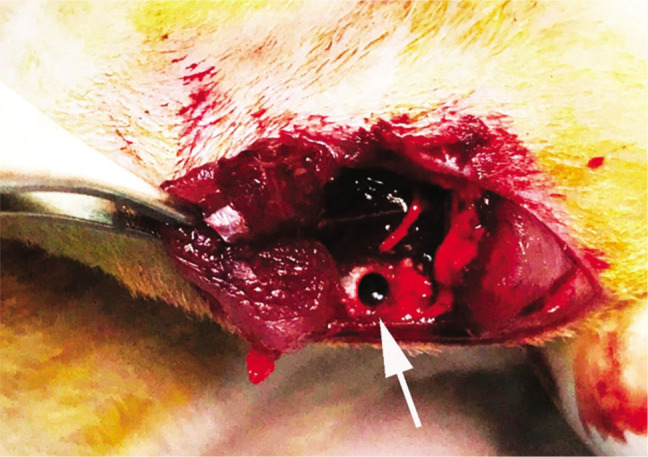
Appearance of the tibia after defect creation (arrow).

Surgery was performed under general anesthesia, administering a mixture of xylazine HCl (Vetaxyl, Vet-Agro®, 20 mg/mL) 12 mg/kg and ketamine (Vetaketam, Vet-Agro®, 100 mg/mL) 75 mg/kg intraperitoneally. After preparation of the site for aseptic surgery, a 2 cm longitudinal skin incision was made overlying the anteromedial part of the proximal tibia medial section of the tibia metaphysis of both legs. The overlying muscle was separated by blunt dissection. The periosteum was mechanically stripped from the proximal tibiae. A 3 mm monocortical hole defect was perpendicularly drilled using a 10,000 rpm cylindrical burr. The standardized hole was unilateral, 3 mm in diameter and 2 mm deep, and was flushed with a copious amount of saline during drilling (**Figure 3**). After the drill hole defect was filled in appropriate groups, the soft tissue was closed with 5–0 absorbable sutures (Vicryl Rapide, Ethicon® Polyglactin 910) using the simple interrupted suture technique.

After the last stitch, enrofloxacin 10 mg/kg (Vicryl Rapide, Ethicon® Polyglactin 910) as an antibiotic and meloxicam 1 mg/kg (Maxicam, Sanovel®) as an analgesic were administered subcutaneously. Animal surgical procedures were performed by the same surgeon to maintain uniformity.

### Histopathological method

All the rats were weighed and then anesthetized by intraperitoneal injection of 90 mg/kg ketamine (Alfamin, Alfasan, IBV) and 10 mg/kg xylazine (Alfazin, Alfasan, IBV). The animals were humanely euthanized by exsanguination. For confirming death, lack of pulse, breathing, corneal reflex, response to firm toe pinch, inability to hear respiratory sounds and heart beat by use of a stethoscope, and graying of the mucous membranes were checked. Then, tibiae were bilaterally removed at 15 d and 30 d after the surgical procedures. After removal of soft tissues, tibiae including the implanted material were fixed in a 10% neutral-buffered formalin solution. After 2 d, the fixated tibial samples were decalcified in a 5% formic acid solution for 1 week, and then tissues were routinely processed by an automatic tissue processor equipment (Leica ASP300S; Leica Microsystems) and embedded in paraffin. Then, 5 μm serial sections were taken from each sample using a rotary microtome (Leica RM 2155) and one of the sections from each rat stained with hematoxylin and eosin (HE) for light microscopy analysis.

The area of total augmentation (TA; mm^2^) and the area of NB (mm^2^) were measured [22]. The numbers of osteoclasts and osteoblasts on the lesioned alveolar bone were counted in an area 1.23 mm^2^ (×400 magnification) [23]. The lesioned areas were analyzed in each rat using a light microscope (Olympus CX41). The Database Manual CellSens Life Science Imaging Software System (Olympus Corporation) was used for histomorphological analysis. Histopathological analyses were performed by a pathologist blinded to the group.

### Immunohistochemical method

Selected sections were stained to demonstrate the presence of bone morphogenetic protein 2 (Anti-BMP2 antibody [ab14933], Abcam), collagen 1 (Collagen 1 antibody [ab34710], Abcam), osteocalcin (OST) (Anti-osteocalcin antibody [ab93876], Abcam), and vascular endothelial growth factor (VEGF [C-1] antibody, sc-7269, Santa Cruz Biotechnology, Inc.) using the streptavidin–biotin peroxidase technique (SBPT), according to the manufacturer’s instructions. All antibodies used a 1/100 dilution. The Mouse and Rabbit Specific horseradish peroxidase / 3,3′-diaminobenzidine (HRP/DAB) Detection Kit – Micropolymer (ab236466) was used as the secondary antibody, and 3,3′-diaminobenzidine was used as chromogen. The primary antibody step was omitted in negative controls. Seven serial sections were prepared and examined for each rat, and 2 areas of each section were examined. All the slides were analyzed for immunopositivity, and a semiquantitative analysis was carried out as detailed later. Samples were analyzed by examining 4 different sections in each sample, which were then scored from 0 to 3, according to the intensity of staining (0, absence of staining; 1, slight; 2, medium; and 3, marked).

After the conventional microscopic examination, computer-assisted histomorphometric measurements and immunohistochemical scoring were obtained using an automated image analysis system (Olympus CX41). The Database Manual CellSens Life Science Imaging Software System (Olympus Corporation) was used for evaluation of the lesioned area.

### Statistical analysis

The sample size was calculated using the G Power 3.1.9.7 program [24] using the following parameters: effect size = 0.6, alpha = 0.05, expected power (1-beta) = 0.80, and number of groups = 5. Although there were 40 samples in total to begin with, it was determined that it would be necessary to account for the probability of loss during the experiment, and accordingly, the choice was made to include 2 additional animals in each group; thus, the total number of samples for use in the analyses amounted to 5 × 10 = 50.

The one-way analysis of variance test was used to determine significant differences between the groups. To determine differences between groups in the histomorphic data analyses, the Duncan multiple comparison test was used. The Kruskal–Wallis test was used to identify whether there were any notable differences in the groups’ immunohistochemical analyses. Pairwise comparisons using the Dwass–Steel–Critchlow–Flinger formula were performed to identify differences across groups. Calculations were made using the SPSS 15.0 program. *P* < 0.05 was set as the level of significance.

## Results

**Figure 4.**
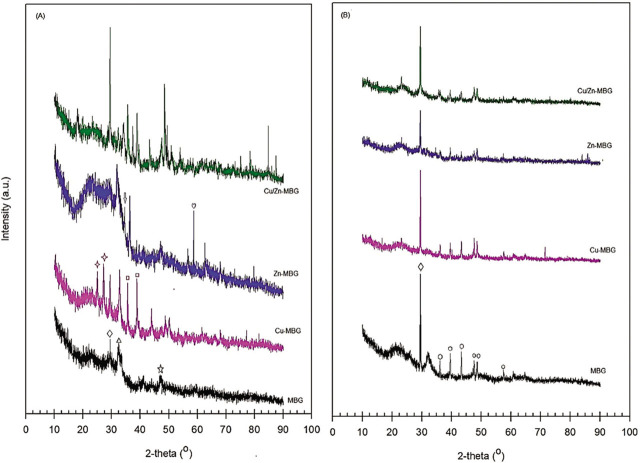
XRD patterns of the MBG and Cu-MBG, Zn-MBG, and Cu–Zn-MBG samples **(A)** before and **(B)** after soaking in SBF for 28 d: □, CaCO_3_, Δ, Ca_2_SiO_4_, 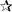, Ca_3_(PO_4_)_2_, 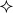, CaSiO_3_, 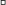, CuO, 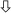, Zn_2_SiO_4_, 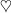, ZnCO_3_, and °, Ca_10_(PO_4_)_6_(OH)_2_. MBG, mesoporous bioactive glass; SBF, simulated body fluid; XRD, X-ray diffraction.

The X-rays patterns of the MBG, Cu-MBG, Zn-MBG, and Cu–Zn-MBG samples before and after soaking in SBF for 28 d are indicated in **Figure 4**. Before soaking, in the case of the XRD pattern of MBG without metal loading, low intensity peaks were observed because of the inclusion of phases of calcium carbonate (CaCO_3_, 2θ = 29.49°), calcium silicate (Ca_2_SiO_4_, 2θ = 32.5°), and calcium phosphate (Ca_3_(PO_4_)_2_, 2θ = 47.3°). In addition to these peaks, the peaks due to Zn_2_SiO_4_ (willemite) at 2θ = 34.5° and ZnCO_3_ at 2θ =58.69° were observed in the zinc loaded product [25]. On the other hand, for the products including only copper or zinc loaded products with copper, new peaks were observed at 2θ = 25.0° and 27.1° and at 2θ = 35.59° and 38.8°, originating from the CaSiO_3_ (in wollastonite) and CuO (in tenorite) phases, respectively. These results show that carbonated and phosphated calcium silicate bioactive glasses were formed. Additionally, when the XRD patterns of metal loaded products are compared with those of MBG, the presence of new peaks in the former indicates that these metals have settled in the structure of bioactive glasses.

After soaking in SBF for 28 d, some characteristic peaks of the calcite, larnite, and wollastonite phases were preserved and the calcite peak intensity increased significantly. Additionally, the peaks at 2θ = 36.07°, 39.51°, 43.25º, 47.53°, 48.66º, and 57.92º caused by hydroxyapatite (Ca_10_(PO_4_)_6_(OH)_2_) were observed in all the samples [26]. It was observed that the intensity of these peaks was found to be lower in metal loaded products.

**Figure 5.**
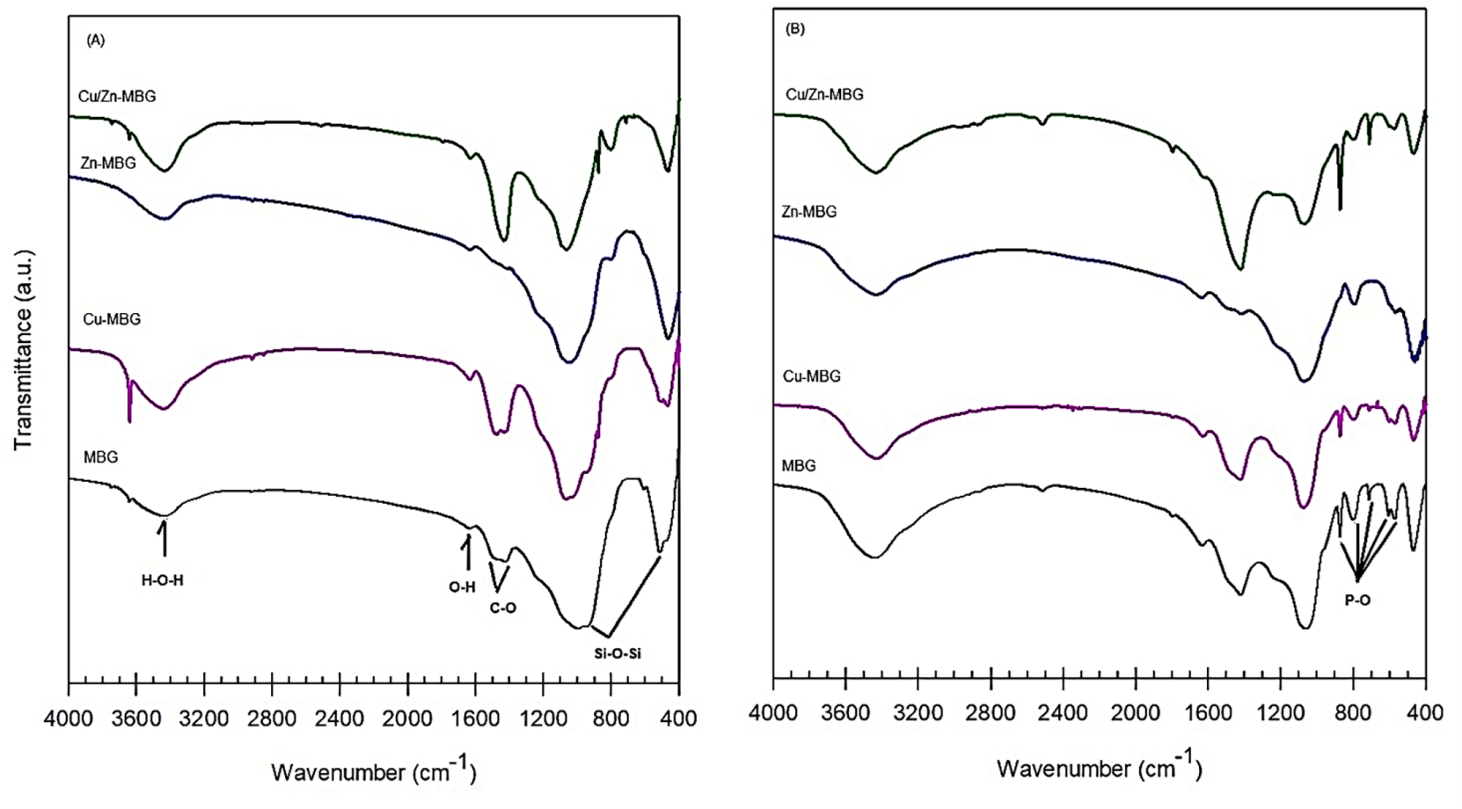
FTIR spectra of the MBG, Cu-MBG, Zn-MBG, and Cu–Zn-MBG samples **(A)** before and **(B)** after soaking in SBF for 28 d. FTIR, Fourier transformed infrared; MBG, mesoporous bioactive glass; SBF, simulated body fluid.

FTIR spectra in the wavelength range 4000–400/cm of MBG, Cu-MBG, Zn-MBG, and Cu–Zn-MBG samples, obtained before and after soaking them in SBF, are shown in **Figure 5**. When the FTIR spectra of MBG and metal loaded bioactive glasses are examined before soaking them in SBF, it is seen that the spectra of the metal loaded samples are similar to that of the MBG sample despite small changes in the region of the low wave number (1200–400/cm) range. For MBG, bands resulting from asymmetric and symmetrical Si–O–Si stretching vibration are observed at wavelengths of approximately 997/cm and 473/cm [27].

Furthermore, the peak of water bending vibration caused by surface hydroxyl groups, the peak originating from water adsorbed to the surface, and the peak caused by carbonate were observed at 1631/cm, at 3437/cm, and in the range of 1422–1491/cm, respectively [26]. For the metal loaded samples, it was observed that while the asymmetric Si–O–Si band shifted to higher wave numbers, and the symmetrical Si–O–Si band to lower wave numbers, their intensities did not change significantly. On the other hand, it was observed that the carbonate band strength increased with Cu and Cu–Zn loading, but not in the product containing Zn. Among the samples soaking in SBF for 28 d, the peaks observed only in the Zn-loaded product at 793/cm and 565/cm and the peaks observed in the others at 873/cm, 793/cm, 713/cm, 568/cm, and 603/cm indicate the formation of hydroxyapatite on the surface of bioactive glasses [27,28,29,30].

**Figure 6.**
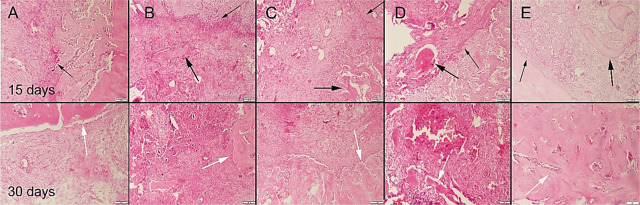
Histopathological appearance of the groups on the 15th and 30th days after graft application in the **(A)** control, **(B)** MBG, **(C)** Cu-MBG, **(D)** Zn-MBG, and **(E)** Cu–Zn-MBG groups. Defect areas (thin arrows), graft materials (thick arrows), and newly formed bone areas (white arrows), HE, bars = 100 μm. MBG, mesoporous bioactive glass.

**Figure 7.**
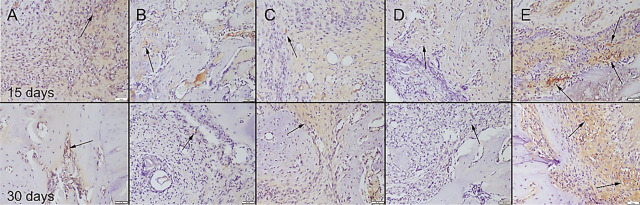
Collagen 1 immunohistochemical expression among the groups on the 15th and 30th days after graft application in the **(A)** control, **(B)** MBG, **(C)** Cu-MBG, **(D)** Zn-MBG, and **(E)** Cu–Zn-MBG groups. Positive expressions (arrows) were observed in connective tissue. SBPT, bar = 50 μm. MBG, mesoporous bioactive glass; SBPT, streptavidin–biotin peroxidase technique.

**Figure 8.**
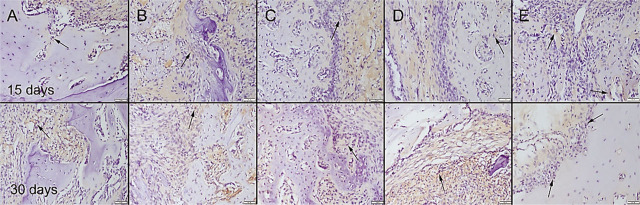
BMP2 immunohistochemical expression among the groups on the 15th and 30th days after graft application in the **(A)** control, **(B)** MBG, **(C)** Cu-MBG, **(D)** Zn-MBG, and **(E)** Cu–Zn-MBG groups. Slight positive reactions (arrows) were observed in some cells. SBPT, bar = 50 μm. MBG, mesoporous bioactive glass; SBPT, streptavidin–biotin peroxidase technique.

**Figure 9.**
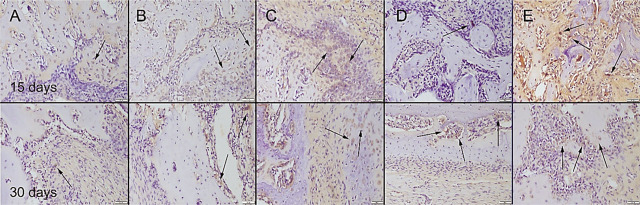
OST immunohistochemical expression among the groups on the 15th and 30th days after graft application in the **(A)** control, **(B)** MBG, **(C)** Cu-MBG, **(D)** Zn-MBG, and **(E)** Cu–Zn-MBG groups. Slight-to-moderate immunopositive reactions (arrows) were observed, especially in osteoclastic cells. SBPT, bar = 50 μm. MBG, mesoporous bioactive glass; OST, osteocalcin; SBPT, streptavidin–biotin peroxidase technique.

**Figure 10.**
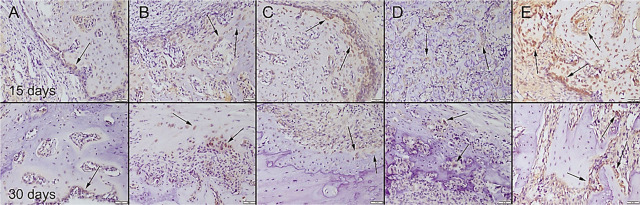
VEGF immunohistochemical expression among the groups on the 15th and 30th days after graft application in the **(A)** control, **(B)** MBG, **(C)** Cu-MBG, **(D)** Zn-MBG, and **(E)** Cu–Zn-MBG groups. Slight-to-moderate immunopositive reactions (arrows) were observed, especially in osteoclastic cells. SBPT, bar = 50 μm. MBG, mesoporous bioactive glass; SBPT, streptavidin–biotin peroxidase technique; VEGF, vascular endothelial growth factor.

During the surgical procedure, defects were produced in both of the 2 tibiae unilaterally in all rats. There was no total fracture occurring in any of the rats and bone integrity was preserved. All rats easily used their legs 1 d after operation. No complication occurred in any of the rats. At the histopathological examination of the bone defect areas, fibrous tissue proliferation was noticed in all groups. There was graft material observed in all groups except the control group in the 15th-day group. Bone formation was noticed in the 30th-day group in all groups. Bone formation was more prominent in the Cu–Zn-MBG group (**Figure 6**). Collagen I immunohistochemistry findings revealed that the most marked increase in expression was observed in the Cu–Zn-MBG group (**Figure 7**). At the BMP2 immunohistochemical examination, slight expressions were observed in all groups. Bone formations were most marked in the Cu–Zn-MBG group (**Figure 8**). At the OST immunohistochemical examination, slight-to-moderate expressions were observed in all groups. Expressions were most marked in the Cu–Zn-MBG group in bone cells, especially in osteolastic cells (**Figure 9**). At the VEGF immunohistochemical examination, slight-to-moderate expressions were observed in all groups. Expressions were most marked in the Cu–Zn-MBG group in bone cells, especially in osteolastic cells (**Figure 10**). The histomorphological results are shown in **Table 1** and the statistical analysis results of immunohistochemical scores are shown in **Table 2**.

**Table 1.** Statistical analysis results of histomorphic data between the groups

**Groups**	**15 days**	**30 days**
**Total augmentation area (mm^2^)**		
Control	2.04 ± 0.46	2.44 ± 0.31
MBG	7.04 ± 0.78†	8.04 ± 1.35†
Cu-MBG	8.84 ± 0.87†	11.74 ± 1.37‡
Zn-MBG	10.20 ± 0.34§	12.58 ± 1.64‡
Cu/Zn-MBG	12.54 ± 0.81§	14.44 ± 0.89§
**Defect closure rate (%)**		
Control	28.20 ± 4.39	36.40 ± 4.03
MBG	44.10 ± 3.90†	55.20 ± 5.30†
Cu-MBG	59.20 ± 3.91‡	75.60 ± 5.08‡
Zn-MBG	64.50 ± 5.72‡	77.20 ± 5.20‡
Cu/Zn-MBG	74.80 ± 4.77§	93.70 ± 2.75§
**New bone area (mm^2^)**		
Control	0.19 ± 0.03	1.54 ± 0.43
MBG	0.31 ± 0.03	1.77 ± 0.47†
Cu-MBG	1.29 ± 0.08†	2.61 ± 0.37‡
Zn-MBG	1.24 ± 0.13†	2.35 ± 0.15‡
Cu/Zn-MBG	1.91 ± 0.10§	3.80 ± 0.14§
**Residual material area (mm^2^)**		
Control	0.00 ± 0.00	0.00 ± 0.00
MBG	17.80 ± 0.78†	7.60 ± 0.96†
Cu-MBG	17.30 ± 1.41†	7.30 ± 1.41†
Zn-MBG	15.80 ± 2.44‡	8.60 ± 1.42†
Cu/Zn-MBG	12.90 ± 3.54§	5.50 ± 1.26§
**Osteoclast number**		
Control	6.30 ± 1.41	9.00 ± 1.49
MBG	12.30 ± 1.70†	16.80 ± 1.61†
Cu-MBG	16.60 ± 2.06‡	20.60 ± 1.57‡
Zn-MBG	17.10 ± 1.59‡	23.30 ± 2.40‡
Cu/Zn-MBG	19.70 ± 1.05§	29.20 ± 2.89§
**Osteoblast number**		
Control	10.10 ± 0.99	20.40 ± 1.17
MBG	13.70 ± 2.66†	25.20 ± 2.27†
Cu-MBG	16.90 ± 1.66‡	29.20 ± 1.75‡
Zn-MBG	16.90 ± 2.23‡	27.10 ± 2.07‡
Cu/Zn-MBG	24.30 ± 1.88§	33.00 ± 1.56§

Data presented by mean ± standard deviation (SD). One-way ANOVA Duncan test was used in statistical analysis. The difference of mean between group were tested by one-way ANOVA (with a Duncan post hoc test).

† *P* < 0.05 compared the Control;

‡ *P* < 0.01 compared the Control;

§ *P* < 0.001 compared the Control.

**Table 2.** Statistical analysis results of immunohistochemical scores between the groups

	**Col-1**	**BMP-2**	**OST**	**VEGF**
**15 days**				
Control	0.50 ± 0.16	0.60 ± 0.51	0.20 ± 0.13	1.20 ± 0.42
MBG	1.10 ± 0.56†	1.20 ± 0.63†	1.20 ± 0.63†	1.50 ± 0.52
Cu-MBG	1.50 ± 0.52†¥	1.30 ± 0.48†	1.30 ± 0.48†	1.80 ± 0.42†
Zn-MBG	1.20 ± 0.42†	1.60 ± 0.51§	1.30 ± 0.48†	1.60 ± 0.51
Cu/Zn-MBG	2.30±0.48§фψδ	1.60 ± 0.69§	1.80 ± 0.91§#ψф	2.30 ± 0.82§¥ψф
**30 days**				
Control	0.70 ± 0.48	1.00 ± 0.47	0.80 ± 0.42	1.50 ± 0.52
MBG	1.40 ± 0.51†	1.80 ± 0.63†	1.60 ± 0.51†	2.10 ± 0.56†
Cu-MBG	1.90 ± 0.31§¥	2.20 ± 0.91‡	1.90 ± 0.56†	2.00 ± 0.47†
Zn-MBG	1.60 ± 0.51†	2.20 ± 0.63‡	1.80 ± 0.78†	2.20 ± 0.63†
Cu/Zn-MBG	2.50 ± 0.52§ф	2.40 ± 0.84§	2.30 ± 0.67§	2.80 ± 0.42§ψ

Data presented by mean ± standard deviation (SD). Kruskal-Wallis test was used in statistical analysis. The difference of mean between group were tested by Dwass-Steel-Critchlow-Flinger pairwise comparisons test.

† *P* < 0.05 compared the Control;

‡ *P* < 0.01 compared the Control;

§ *P* < 0.001 compared the Control.

¥ *P* < 0.05 compared the MBG;

# *P* < 0.01 compared the MBG;

ф *P* < 0.001 compared the MBG.

ψ *P* < 0.05 compared the Cu-MBG.

δ *P* < 0.05 compared the Zn-MBG.

## Discussion

In this study, MBG, Cu-MBG, ZnMBG, and Cu–Zn-MBG were used for the repairing of surgically induced tibial defects in rats. The results indicated that bioactive glass materials were more effective for bone healing compared with the empty control defects. In addition to better amelioration of the bone defect compared with the control treatment, no tissue reaction to the material was observed to arise from the use of these materials, as ascertained at both the 15th and 30th days post the defect-inducing surgery. The most marked amelioration was observed in the Cu–Zn-MBG administered group.

Biomaterials are synthetic or natural substances that can be used to repair or restore living tissue functions in the body [31]. Due to the increase in instances of injury arising from factors such as automobile crashes or traffic accidents, the impact of bone injury and diseases has increased tremendously over the past few decades. Bioactive glasses have excellent osteoconductivity and biocompatibility, making them suitable for bone regeneration. Research and studies on various kinds of bioactive glasses provide insight into the necessity to adopt multidisciplinary approaches involving various scientific fields to enable this biomaterial to reach its full potential for use as a scaffold that would enable rapid recovery from bone defects. MBG materials, a new generation of bioactive glass, are developed with higher specific surface area and control over the mesoporous structure, thus offering a new, promising material for bone regeneration [32]. These study findings lend credence to the idea that bioactive glass may have the potential to bring about a significant improvement in the medical technology used to remedy bone defects.

There is an increasing need for the development of effective and synthetic scaffolds for the repairing of major bone defects caused by malignancy, trauma, congenital diseases, or any combination of these [33]. The gold standard for the treatment of bone defects is autologous bone graft materials; however, this option is beset with problems, such as donor site morbidity and limited supply for regeneration. Bone allografts may offer another alternative means, but are characterized by a risk of disease transmission, uncertainty concerning final acquisition of the outcome of bone healing, and adverse host immune reaction; moreover, they are very expensive [34]. The results of the present study indicate that one of the options for this material could be MBS, specifically Cu–Zn-MBG, which enables a significant improvement in bone defects.

Copper (Cu) ions have played a pivotal and direct role in the stimulation of angiogenesis [35]. Copper ions provide proliferation and migration of endothelial cells in a dose-dependent manner [36] and promote wound healing through upregulation of VEGF expression in stimulated cells [37]. The results of this study also showed that Cu and Zn caused better healing compared to the control group, and the Cu–Zn combination supported the healing better compared to any one of these materials being used by themselves.

The osteogenic property of bioactive glass particles may be related to the activation of an autocrine mechanism in osteoblasts [38]. Bone remodeling is an important process for maintaining normal skeletal structure and function. Many cell types and factors play a role in the occurrence of the bone remodeling process. Osteoclasts and osteoblasts are the 2 main cell types that play important roles in this process [39]; osteoclasts are responsible for aged bone resorption while osteoblasts are responsible for NB formation. Under normal physiological conditions, resorption and formation are equal and stable [40]; in conformity with this general observation, the findings from the present study have demonstrated that MBG caused a significant increase in osteoblastic and osteoclastic activity.

This study has some limitations. First, the number of animals in the groups was kept as low as possible, since the study was carried out in 2 stages and involved using a large quantum of material. Second, radiological and advanced molecular techniques were not used in the study.

## Conclusion

The study findings demonstrate that MBG, Cu-MBG, Zn-MBG, and Cu–Zn-MBG are effective in bone defect healing. A better amelioration was observed in the Cu–Zn-MBG administered rats. This study also revealed that these MBG materials can be used to increase the effectiveness and speed of bone healing. Further studies are needed to evaluate whether these materials are appropriate for use in treating bone defects in humans.
